# Editorial: Mindfulness and resilience in the digital age: addressing cyberattacks and enhancing mental health and performance among adults

**DOI:** 10.3389/fpubh.2025.1671991

**Published:** 2025-08-18

**Authors:** Ali Nawaz Khan, Naseer Abbas Khan, Ali Ahmad

**Affiliations:** ^1^Research Center of Hubei Micro & Small Enterprises Development, School of Economics and Management, Hubei Engineering University, Xiaogan, China; ^2^Malik Firoz Khan Noon Business School, University of Sargodha, Punjab, Pakistan; ^3^Department of Business Administration, Nijmegen School of Management, Radboud University, Nijmegen, Netherlands

**Keywords:** mental health, mindfulness, cyberattacks, performance, resilience, social media, adults

## Introduction

In today's digital era, the integration of social media and digital platforms has become an essential component of adult life (1). While the rapid adoption of digital tools has revolutionized communication, education, entertainment, and personal development (2), it has also increased individuals' exposure to cyberattacks, cyberbullying, and various psychological threats (3). This Research Topic explores how psychological wellbeing is influenced by digital threats and considers the protective role of mindfulness and resilience.

Modern adults—especially university students and working professionals—face heightened psychological stress due to their dependence on digital media (4). Despite the numerous benefits of digital connectivity, these platforms have become channels for harmful practices such as cyberbullying, identity theft, and digital harassment. Such experiences contribute to increased psychological distress, diminished academic or professional performance, and reduced overall life satisfaction. This issue compiles multidisciplinary empirical research to better understand and counteract the harmful effects of digital engagement through psychological strengths like mindfulness and resilience.

## Synthesis of research findings

AlQahtani et al. present strong evidence of the correlation between social media addiction and negative body image among Saudi Arabian adult females. That 71.2% of the participants presented negative body image, especially those with social media addiction and non-normal BMI levels, highlights the strong psychological influence of social media use. Interestingly, their research came up with the fact that beauty filter use did not correlate with adverse body image, refuting some earlier hypotheses surrounding digital enhancement tools.

Tu et al. made important contributions to the intricate social media usage and psychological wellbeing relationship. Their results illustrate that whereas social media use positively affects positive affect, mindful practice has more integrated benefits, having a positive effect on positive affect and also lowering negative affect. The study specifically underscores the buffer role of mindfulness against psychological distress with a total effect of −0.11.

Zhu et al. enlightens the interplay between self-esteem, social anxiety, and internet addiction. Their observation that self-esteem has a negative correlation with internet addiction and social anxiety has a positive correlation offers critical information for intervention programs. The findings of the age- and gender-specific analyses are strong across various population groups, indicating the ubiquitous nature of the relationships.

Gan et al. investigated the multifaceted process of online gaming behavior, uncovering fascinating trends in the association between online disinhibition and gaming aggression. Their discovery that moral disengagement acts as a mediator between online disinhibition and aggressive gaming behavior sheds significant light on the psychological processes involved in problem gaming behavior. The investigation into gender trait moderations provides significant considerations in the direction of interventions.

Abate et al. offers detailed evidence on the establishment of resilience through adversity and the efficacy of multiple intervention types. Their observation that resilience-promoting interventions have the largest pooled effect (0.42) among intervention types provides robust evidence in favor of specific resilience-building programs. The research also identifies an important role for multiple protection factors, with self-regulation having the largest effect.

Li et al. made significant contributions to understanding the role of mindfulness in sport training, specifically obsessive passion and cognitive state anxiety. What they found was that mindfulness training for young athletes can assist them in regulating their emotional states in a better manner and preventing maladaptive obligatory exercise behaviors. Fan et al. revealed concerning statistics about mental health among university students, with 24.54% showing suboptimal mental health conditions. Their identification of eight significant predictors of mental health, including psychological resilience and family support, provides valuable guidance for developing targeted support systems in educational settings.

The study by Ye et al. provides valuable information regarding the psychological processes of cyberbullying behaviors. Their finding that overconfidence and excess moral sense were positive predictors of cyberbullying behaviors, and the startling correlation between cyberbullying and perceived value, presents the need for targeted interventions at these psychological processes.

## Conclusion

The collection of articles presented in this Research Topic elucidates the complex and bidirectional relationships among psychological resilience, mental wellbeing, and digital interactions. The research identifies resilience and mindfulness as potentially efficacious protective factors against psychological distress emanating from digital environments. There is a pressing imperative for future empirical investigations and scholarly discourse focused on the development and empirical validation of targeted interventional strategies to address these multifaceted and evolving challenges. This comprehensive body of literature demonstrates the dual nature of technological engagement on adult psychological wellbeing, underscoring the critical importance of fostering adaptive capabilities to effectively manage digital vulnerabilities, including cybersecurity threats. Future research endeavors should employ diverse methodological approaches, incorporating both qualitative investigations and experimental paradigms, to enhance our understanding of digital mental health across developmental stages. Given the expanding trajectory of global digital interconnectedness, equipping individuals with robust psychological mechanisms to navigate digital adversity has transcended mere preference to become an essential imperative for contemporary society.
